# Antimicrobial peptide gene *BdPho* responds to peptidoglycan infection and mating stimulation in oriental fruit fly, *Bactrocera dorsalis* (Hendel)

**DOI:** 10.1186/s13568-017-0533-8

**Published:** 2018-01-11

**Authors:** Shi-Huo Liu, Hong-Fei Li, Yang Yang, Dong Wei, Hong-Bo Jiang, Wei Dou, Guo-Rui Yuan, Jin-Jun Wang

**Affiliations:** 1 Key Laboratory of Entomology and Pest Control Engineering, College of Plant Protection, Southwest University, Chongqing, 400716 China; 2 Academy of Agricultural Sciences, Southwest University, Chongqing, 400716 China

**Keywords:** *Bactrocera dorsalis*, Antimicrobial peptide, Phormicin, Immunity, Mating

## Abstract

Phormicins belong to defensin family, which are important antimicrobial peptides (AMPs) in insects. These AMPs are inducible upon challenging by immune triggers. In the present study, we identified the cDNA of a phormicin gene (*BdPho*) in the oriental fruit fly, *Bactrocera dorsalis* (Hendel), a ruinous agricultural pest causing great economic losses to fruits and vegetables. The cDNA of *BdPho* contains a 282 bp open reading frame encoding 93 amino acid residues, and the predicted molecular weight and isoelectric point of BdPho peptide were 9.83 kDa and 7.54, respectively. Quantitative real-time PCR analyses showed that the transcription level of *BdPho* was the highest in adult during different developmental stages and was the highest in abdomen among adult tagmata. Moreover, *BdPho* was highly expressed in fat body among different tissues, both in female and male adult. The mRNA level of *BdPho* was significantly up-regulated to 7.46- and 14.53-fold at 3 and 6 h after the insects were challenged with peptidoglycans from *Escherichia coli* (PGN-EB), respectively, suggesting its antimicrobial activity against Gram-negative microorganisms. Furthermore, the expression level of *BdPho* was significantly up-regulated to 3.83-fold after mating, suggesting that female adults might enhance their immunity by up-regulating the expression level of *BdPho* during mating. These results firstly describe the basic properties of the phormicin gene from *B. dorsalis*, and lay the foundation for investigating functional properties of AMPs and exploring the molecular mechanisms in the immune system.

**Electronic supplementary material:**

The online version of this article (10.1186/s13568-017-0533-8) contains supplementary material, which is available to authorized users.

## Introduction

The natural immune system of insect consists of cellular and humoral responses. The cell-mediated immunity includes phagocytosis, pinocytosis, nodulation, and encapsulation of invading microbes (Schmidt et al. 2001). In contrast, the humoral response includes melanization and the secretion of antimicrobial peptides (AMPs). AMPs are one of the most essential molecules of the humoral immune response and are synthesized mainly in insect fat body (Hoffmann 1995; Hultmark 1993). During the past decades, AMPs have been the focus of intense research on insect immunity and are now regarded as a vital part of the defense system in insects. Insect AMPs are not only involved in the defense against invading microorganisms in the haemolymph, but also regulate the bacterial community homeostasis in host gut (Buchon et al. 2013). It has been reported that the categories and expression levels of AMP genes could affect bacterial resistance of host in *Drosophila* (Unckless et al. 2016) and gut symbiont which could in turn enhance insecticide resistance in *Bactrocera dorsalis* (Cheng et al. 2017). These interesting results broadened our knowledge in pest control and showed that insect AMPs could serve as target or gut-symbiont-killer to control pest indirectly.

Defensins are the largest family of AMPs and are found in almost all organisms including animals, plants and microorganisms. The cysteine-rich polypeptide defensin was first isolated from the flesh fly, *Sarcophaga peregrine* (Matsuyama and Natori 1988). Thereafter, more and more different defensins have been identified from various insects. Insect defensins are widely investigated in the orders of Diptera, Coleoptera, Lepidoptera, Hemiptera, and Hymenoptera (Liu et al. 2017; Tian et al. 2010; Ueda et al. 2005; Wang et al. 2013b; Wen et al. 2009). Note worthily, defensin is also present in the ancient order of Odonata (Bulet et al. 1992), suggesting that insect defensins may derive from a common ancestor gene. Phormicin (also called phormia defensin) is a cysteine-rich antimicrobial peptide belonging to the defensin family. It was firstly identified in *Phormia terraenovae* (Lambert et al. 1989). Phormicins have broad-spectrum antimicrobial activity against both Gram-positive and Gram-negative bacteria, as well as fungi (Lambert et al. 1989; Lamberty et al. 1999, 2001).

The oriental fruit fly, *B. dorsalis* (Hendel) belonging to Tephritidae of Diptera, is one of the most destructive agricultural pests in many regions of the world (Clarke et al. 2005). Dipteran insects are considered as microorganism-rich and expected to be abundant in AMPs. To date, only five groups of AMPs have been identified in this species, including cecropins, defensins, attacins, bactrocerin, and diptericins (Dang et al. 2009; Jiang et al. 2014; Liao et al. 2015; Liu et al. 2017). To better understand the adaptation of *B. dorsalis* to different environments and also provide more natural molecules with potential for designing effective antibiotics applied in medicine and agriculture (Yi et al. 2014), it is worthy to identify more AMPs and analyze the immune response challenged by the immune triggers in *B. dorsalis*. Therefore, we initiated this study to identify a phormicin-encoding gene (*BdPho*) in *B. dorsalis*, and to investigate the response model of *BdPho* to immune-stimulation. The results of this study provided fundamental information for understanding the molecular mechanisms in immune system of Tephritidae insects, and also provide innovative ideas for developing effective insecticides.

## Materials and methods

### Insects

The oriental fruit fly, *B. dorsalis* was originally obtained from Hainan province of China in 2008, and maintained under laboratory conditions at 27.5 ± 0.5 °C, 75 ± 5% relative humidity (RH), and a photoperiod of 14:10 (L:D). Larvae and adults were reared on artificial diets as previously described (Wang et al. 2013a). To obtain virgin adults, newly enclosed adults were sex-biased separated and reared in different cages until collection.

For the spatiotemporal expression profiles of *BdPho*, samples at different developmental stages and different tissues of adult were collected as described previously (Liu et al. 2017).

### RNA isolation and cDNA synthesis

Total RNA of each sample was isolated separately using TRIZOL reagent (Invitrogen, Carlsbad, CA) according to the manufacturer instructions. Before cDNA synthesis, the contaminating genomic DNA of each RNA sample was digested with DNase I (Promega, Madison, WI). First-strand cDNA was synthesized as described previously (Liu et al. 2017).

### Molecular cloning

The specific primers used for amplifying open reading frame of *BdPho* (forward, 5′-TTTCACACACCTCAGTAAACTTTCT-3′; reverse, 5′-TTATTCCCCTCTCTTCAGTCGT-3′) were synthesized by Invitrogen in Shanghai, China. All PCRs were performed with 25 μL reaction mixtures as described previously (Liu et al. 2017). PCR parameters were: 94 °C for 3 min, followed by 35 cycles of 94 °C for 30 s, anneal of 58 °C for 30 s, and extension of 72 °C for 1 min, final extension of 72 °C for 7 min. The purified PCR products were sequenced by Invitrogen.

### Sequence analysis and phylogenetic tree construction

Multiple sequence alignments were conducted using online software ClustalW2 (http://www.ebi.ac.uk/Tools/msa/clustalw2). The theoretical parameters of the deduced protein sequences were computed using the ExPASy Proteomics Server (http://cn.expasy.org/tools/pi_tool.html). The signal peptide and α-helix structures of the BdPho were predicted by the SignalP 4.1 server program (http://www.cbs.dtu.dk/services/SignalP) (Petersen et al. 2011) and SOPMA secondary structure prediction (https://npsa-prabi.ibcp.fr/cgi-bin/npsa_automat.pl?page=/NPSA/npsa_sopma.html) (Combet et al. 2000), respectively. The online software DISULFIND (http://disulfind.dsi.unifi.it/) was used to predict cysteine disulfide bonding state and connectivity of the putative protein. And the tertiary structure of BdPho was predicted by online software SWISS-MODEL (https://swissmodel.expasy.org/) (Biasini et al. 2014).

The phylogenetic analysis on the basis of amino acid sequences was performed with MEGA 6.0 (Tamura et al. 2011) using the Maximum Likelihood (ML) method with 1000 bootstrap tests. The 15 amino acid sequences of phormicins for phylogenetic analysis were from *Rhodnius prolixus*, *Panstrongylus megistus*, *Triatoma infestans*, *T. sordida*, *Apis cerana japonica*, *A. dorsata*, *A. cerana cerana*, *A. mellifera*, *B. dorsalis*, *B. oleae*, *Ceratitis capitata*, *Protophormia terraenovae*, *Sarcophaga peregrina*, *Aedes aegypti*, and *Musca domestica*. All sequences were obtained from NCBI database (http://www.ncbi.nlm.nih.gov).

### Quantitative real-time PCR

Quantitative real-time PCR (qRT-PCR) assay was carried out to investigate the relative mRNA levels of *BdPho* during various developmental stages, body parts, tissues, and female adults treated by immune triggers, as well as virgin females and mated females. The qRT-PCR was performed using GoTaq qPCR Master Mix (Promega) on a Mx3000P thermal cycler (Stratagene, La Jolla, CA). The *α*-*tubulin* (GenBank accession number: GU269902) and *RPS3* (XM_011212815) of *B. dorsalis* were served as internal reference genes based on our previous study (Shen et al. 2012) and *RPS3* was also further validated in this study (Additional file 1: Figure S1, Additional file 2: Figure S2). All qRT-PCRs were performed in a final reaction mixture of 10 μL contained 5 μL qPCR Master Mix, 0.5 mM each of forward and reverse primers, and 0.5 μL cDNA templates (about 600 ng/μL). The PCR parameters were 95 °C for 2 min, followed by 40 cycles at 95 °C for 15 s and 60 °C for 30 s. To verify a single PCR product, the melting curve analysis from 60 to 95 °C after the cycling protocol was conducted. Each qRT-PCR experiment contained 3–4 independent biological replicates and 2 technical replicates. The qRT-PCR primers for *BdPho* (forward, 5′-TACTTTTCGCCGGTCCCAT-3′; reverse, 5′-CCACGTCCATTGCAGTAACC-3′) and for the internal reference genes *α*-*tubulin* (forward, 5′-CGCATTCATGGTTGATAACG-3′; reverse, 5′-GGGCACCAAGTTAGTCTGGA-3′) and *RPS3* (forward, 5′-TGGATCACCAGAGTGGATCA-3′; reverse, 5′-TAAGTTGACCGGAGGTTTGG-3′) were designed using online software Primer 3 (http://primer3.ut.ee/) and synthesized by Invitrogen.

### Immune challenge

The purified peptidoglycan from the Gram-positive bacterium *Staphylococcus aureus* (PGN-SA, InvivoGen, San Diego, CA) and Gram-negative bacterium *E. coli* O111:B4 (PGN-EB, InvivoGen) were used as immune triggers. PGN-EB and PGN-SA powder were diluted with the sterilized endotoxin-free water (InvivoGen) to a final concentration of 100 μg/mL, respectively. Eighty 5-day-old virgin female adult flies were randomly collected and divided into four groups: named PGN-EB group, PGN-SA group, control group, and blank group, respectively. Each adult of PGN-EB group, PGN-SA group, and control group were injected with 200 nL PGN-EB solution, PGN-SA solution, and the sterilized endotoxin-free water, respectively, and no injection for adults in blank group. Three individuals of flies were randomly collected from each group after 3, 6 and 9 h post injection, respectively. Experiments were repeated three times independently and total RNA of each sample was extracted as described above.

### Mating experiment

Female and male adults were sex-biased reared for 9 days after eclosion, respectively. On the 10th day, 20 individuals of each female and male flies randomly collected from corresponding cage and matched in a new cage to allow mating at dark. When female and male flies begin mating, the couples were carefully transferred to small cages and raised for 12 h, avoiding disturbing. Finally, six individuals of each mated and virgin female flies were randomly collected. Total RNA isolation was performed as described above. The experiment was repeated three times independently.

### Data analysis

The relative expression levels were calculated based on the expression level of reference genes by qBase (Hellemans et al. 2007) and data were analyzed with SPSS 19.0 software (IBM, Chicago, IL). Significant differences were analyzed with *t*-test and one-way ANOVA (followed by a Tukey test) for comparison of two means or multiple comparisons, respectively.

## Results

### Sequence and phylogenetic analysis of *BdPho*

**Fig. 1:**
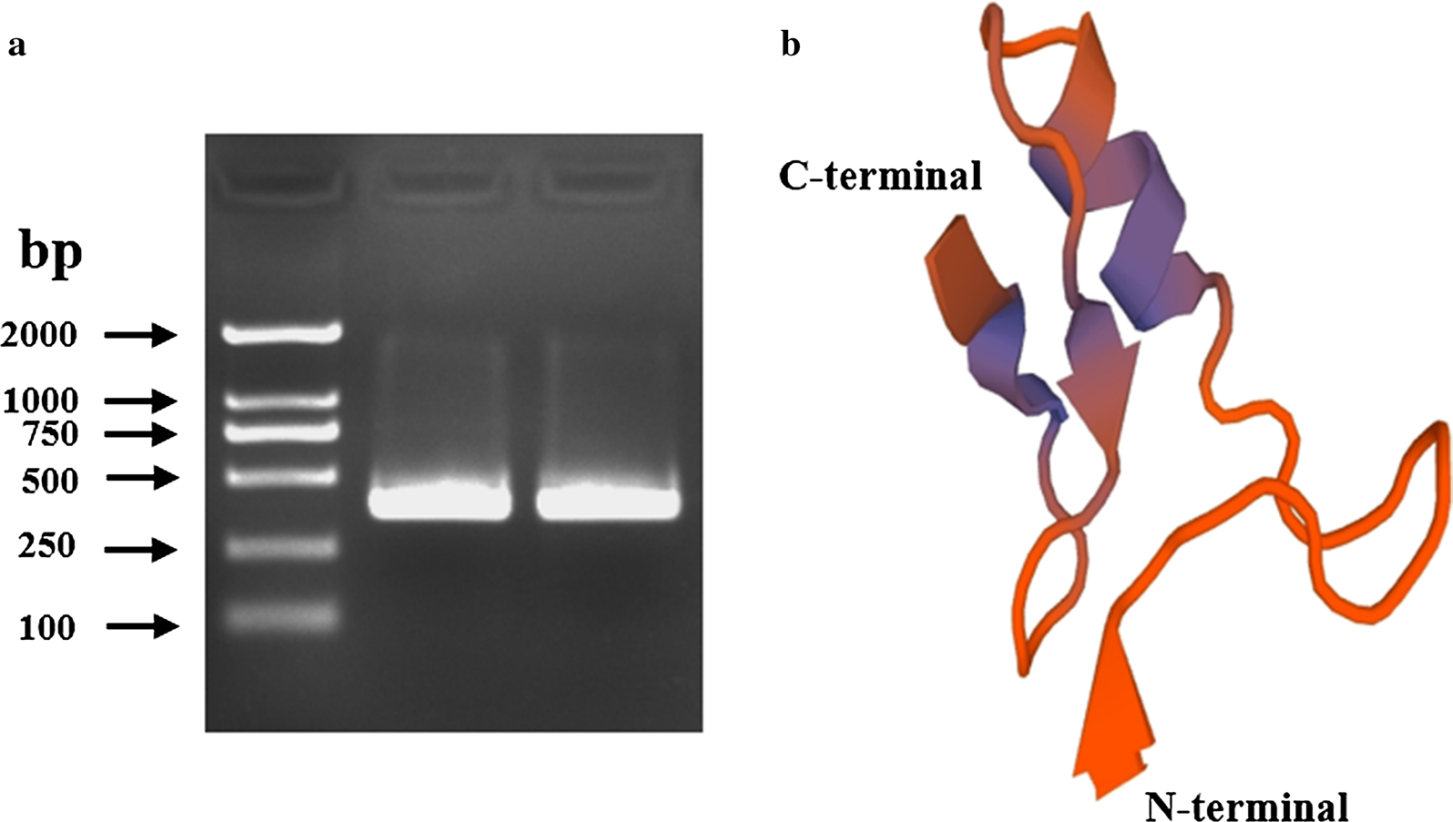
Electrophoresis image of PCR (**a**) and predicted tertiary structure of protein encoded by *BdPho* (**b**)

**Fig. 2:**
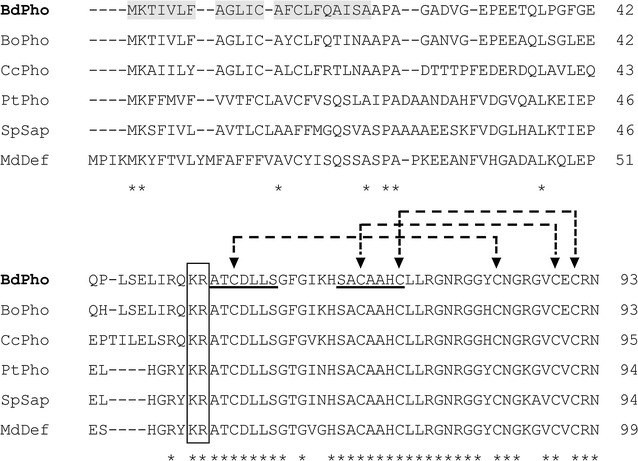
Multiple sequence alignments of amino acid sequence of BdPho with those of other insect defensins. The amino acid sequences are from *B. dorsalis* (BdPho, KY038166), *B. oleae* (BoPho, XP_014095033); *Ceratitis capitata* (CcPho, XP_004537627); *Protophormia terraenovae* (PtPho, P10891); *Sarcophaga peregrina* (SpSap, AAA29984), and *Musca domestica* (MdDef, AIL24687). The signal peptide of BdPho is on a gray background. The conserved mature peptide cleavage sites “-KR-” are boxed, and amino acids corresponding to predicted α-helix are underlined. Identical residues are indicated by * under the residues. The triangles indicate the six conserved cysteine residues and the dashed lines indicate the disulfide bonds

The cDNA fragment of *BdPho* (GenBank accession no. KY038166) was amplified by PCR (Fig. 1a). The 365 bp cDNA sequence has an ORF of 282 nucleotides encoding a protein of 93 amino acid residues. The predicted molecular mass and isoelectric point of BdPho peptide were 9.83 kDa and 7.54, respectively. The predicted tertiary structure showed that BdPho contained a conserved “αββ” structure (Fig. 1b) that is widely distributed in insect defensin family. The first 22 amino acids at the N-terminus of BdPho were predicted as a signal peptide (Fig. 2), suggesting that phormicin in *B. dorsalis* is a secretory peptide. Two α-helix regions and three cysteine disulfide bonds were found at the mature peptide region of BdPho (Fig. 2).

**Fig. 3:**
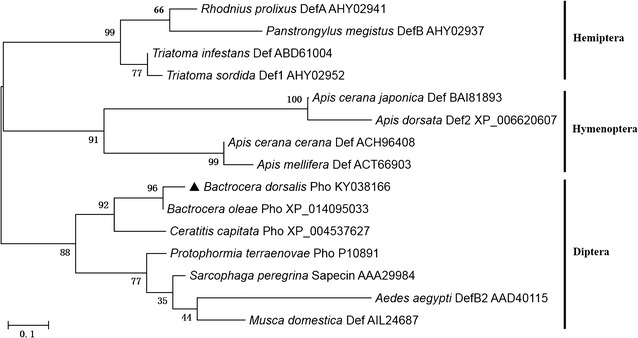
Phylogenetic analysis of 15 insect phormicins and defensins. The phylogram of 15 insect defensin amino acid sequences was generated in MEGA 6.0 using Maximum Likelihood method. Scale bar = 0.1 substitution/site. Bootstrapping analysis was performed 1000 replications. Accession numbers were labeled together with scientific name

The phylogenetic analysis showed that phormicins in dipteran, hemipteran, and hymenopteran species were separated into three corresponding clades (Fig. 3). The BdPho was closely related to the phormicin of olive fruit fly (*B. oleae*) with a bootstrap value of 96%.

### Spatiotemporal expression profiles of *BdPho*

**Fig. 4:**
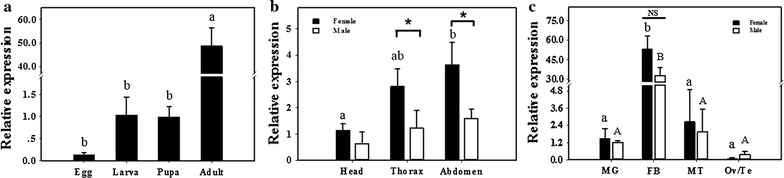
The spatiotemporal expression profiles of *BdPho* in *B. dorsalis*. **a** The relative mRNA expression profiles of *BdPho* in egg, third instar larva, 5-day-old pupa and 5-day-old adult; **b** The relative mRNA expression profiles of *BdPho* in various body parts including head, thorax and abdomen; **c** The relative mRNA expression profiles of *BdPho* in different tissues including Malpighian tubule (MT), fat body (FB), mid gut (MG), ovary (Ov) and testis (Te). Data are presented as mean ± SE (*n* = 4). Lowercase letters above black bars and uppercase letters above white bars indicate statistical difference by ANOVA followed by the Tukey’s multiple comparison test (*P* < 0.05), respectively. Significant differences between female and male determined with *t*-test, asterisks indicate significant differences in relative expression (*P < 0.05; **P < 0.01), and NS means no significant differences

The results of developmental expression profiles of *BdPho* showed that the highest and lowest mRNA levels occurred in adult and egg, respectively (Fig. 4a), besides, the mRNA level in adult stage was significantly higher than that in egg stage. As for adult body parts, the highest mRNA level of *BdPho* was found in the abdomen in both females and males, but the significant difference was only found between abdomen and head in females. The relative expression of *BdPho* in thorax and abdomen of female adult was significantly higher than that in male adult (Fig. 4b). For different tissues, the highest mRNA level of *BdPho* was observed in the fat body compared with those in Malpighian tubule and midgut, regardless of the sex (Fig. 4c).

### Transcriptional response of *BdPho* to immune challenge and mating

**Fig. 5:**
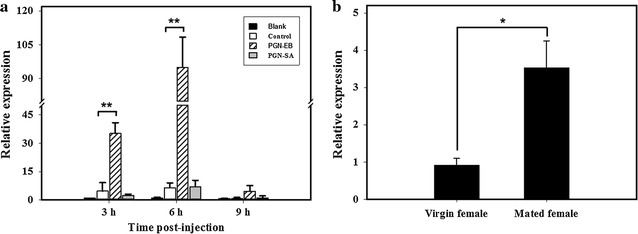
Effects of PGN challenge and mating on the mRNA expression levels of *BdPho* in *B. dorsalis*. **a** Relative mRNA levels of *BdPho* after PGN-EB and PGN-SA challenge. Blank: without injection; Control: injected with 200 nL sterile endotoxin-free water; PGN-EB: injected with 200 nL PGN-EB solution; PGN-SA: injected with 200 nL PGN-SA solution. **b** Relative mRNA levels of *BdPho* after mating stimulation. Data represent mean relative level values (mean ± SE) to reference genes of three independent biological replicates. The two-tailed, unpaired *t*-test was used to test significance. Asterisks indicate significant differences in relative expression (*P < 0.05; **P < 0.01)

As shown in Fig. 5a, the relative mRNA levels of *BdPho* significantly increased up to 7.46- and 14.53-fold at 3, and 6 h after PGN-EB injection compared to water injection, respectively. The expression of *BdPho* was also up-regulated at 9 h post PGN-EB injection, but without significant differences. The injection of PGN-SA did not show significantly induction of *BdPho* at 3, 6 and 9 h post injection (Fig. 5a). The relative mRNA level of *BdPho* significantly increased up to 3.83-fold at 12 h after starting mating compared to that of virgin females (Fig. 5b).

## Discussion

In the present study, we identified the cDNA sequence of a gene encoding phormicin in *B. dorsalis*. This phormicin belongs to the insects defensin family which are small cationic peptides and full of cysteines. Usually, defensins contain three peptide regions: signal peptide, prodefensin, and the mature peptide. As previously reported, the cleavage recognition sites between prodefensin and mature peptide were ‘-KR-’ (Lowenberger et al. 1999). BdPho peptide also contained this conserved ‘-KR-’ cleavage site. Multiple sequence alignments showed that BdPho has a high degree of identity (87.5–97.5%) with other insect phormicins and defensins in mature peptides, particularly the six conserved cysteine residues, which form three disulfide bridges. These three disulfide bonds adopted a ‘cysteine stabilized αβ’ (CS αβ) scaffold (Cornet et al. 1995), and the predicted tertiary structure also showed this conserved “αβ” structure in BdPho (Fig. 1b). The predicted pairing patterns of these six conserved cysteine residues were the same as that of BdDef (C1–C4, C2–C5 and C3–C6) (Liu et al. 2017), and such strong conservation suggested that these cysteines and tertiary structures might have an important structural function in these proteins.

Previous studies demonstrated that the net positive charges carried by AMPs were associated with antimicrobial activities against diverse microbes (Wang et al. 2013b). Although different AMPs have different mechanisms and modes of action, the electrostatic attraction between the cationic AMPs and negatively charged microbial membranes was the first step of the interaction between AMPs and microbes (Yeung et al. 2011). In this study, we found that BdPho mature peptide possesses + 2.9 charges at pH 7.0 condition. Depending on these surplus positive charges, BdPho may easily react with negatively charged microbial membranes by electrostatic attraction.

Different insects can produce different AMPs, and the transcription levels of particular AMPs differ a lot among species of insect. Between different developmental stages, tagmata, and tissues of a specific insect, expression profiles of AMPs are variable. For instance, *BmDefA* is highly expressed in early pupa and adult stages of *Bombyx mori* (Wen et al. 2009). Similarly, a high level of constitutive expression of *DmDef* was found in pupa and adult stages of *D. melanogaster* (Dimarcq et al. 1994). However, in our previous study, *BdDef* was highly expressed in larva and adult stages of *B. dorsalis* (Liu et al. 2017). In contrast to *BdDef*, *BdPho* was highly expressed in adult stage, but lowly expressed in egg, larva and pupa stages. Although *BdDef* and *BdPho* belong to the same defensin family, their different expression profiles probably suggest a different functional involvement in the immune system during the development of *B. dorsalis*. The results also indicated that *BdPho* might play important roles in fighting against invading microbes in the adult stage.

AMPs are expressed constitutively or induced by microorganisms infection (Lemaitre and Hoffmann 2007). Most insect AMP genes are mainly expressed in immune-related organs such as the fat body, midgut and haemolymph (Wang et al. 2006). Meanwhile, the induction of different insect AMPs, as well as induction in different tissues including fat body, reproductive tissues, midgut, and salivary gland in various insects have been reported (Liao et al. 2015; Tzou et al. 2002; Wang et al. 2013b). Furthermore, it has been well documented that the expression profiles of AMPs in different tissues are related to the category of AMP genes with variations across insect species. In *B. mori*, *BmDefA* was highly expressed in the hemocyte, silk gland and ovary, and lowly expressed in the fat body, Malpighian tubule and midgut (Wen et al. 2009). In contrast, *BtDef* was highly expressed in midgut and salivary, and expressed at a relatively lower level in the ovary and fat body in *Bemisia tabaci* (Wang et al. 2013b). In the current study, *BdPho* was expressed at a significantly high level in fat body, and at a much lower level in midgut, Malpighian tubule, testis and ovary. These results suggest that *BdPho* undertake its roles mainly in the fat body of adults to protect *B. dorsalis* from pathogens infection.

Though some AMPs failed to be induced in *Drosophila* (Hanson et al. 2016), injection of PGN has been considered as an effective way to investigate insect immune response (Neyen et al. 2014). The time course of transcription of *BdPho* was determined at 3–9 h after injection with two kinds of PGNs in female adult of *B. dorsalis*. The transcription level of *BdPho* was significantly induced at 3 h after PGN-EB challenge and reached a higher level at 6 h post injection; while injection of PGN-SA could not significantly stimulate the expression of *BdPho* from 3 to 9 h post injection. These findings implied that *BdPho* could be sharply induced by Gram-negative PGN but weakly induced by Gram-positive PGN. The pattern of induction of *BdPho* is similar with that of *BdDef* in *B. dorsalis* (Liu et al. 2017) and *Mdde* in *M. domestica* (Wang et al. 2006). The *BdDef* was also strongly induced by PGN-EB at 3 and 6 h, but the difference was that *BdDef* could be significantly induced by PGN-EB at 9 h and PGN-SA at 6 h. Though *BdPho* and *BdDef* belong to the same family and have similar expression profiles in tagmata and tissues of *B. dorsalis*, the different induction patterns suggested that *BdPho* and *BdDef* possess different response intensity under the immune challenge of invaded bacteria. Similarly, the transcription of the *Mdde* was increased at 3 h post injection of bacteria, and the peak appeared at 36 h post challenge with Gram-negative bacterium (*E. coli*), while at the same time, injection of *S. aureus* (Gram-positive) did not induce expression of *Mdde* from 3 to 48 h. On the other hand, some differences occurred between the induction of *BdPho* by PGN-EB and the induction of *Mdde* by *E. coli*. For instance, infection of *E. coli* could strongly induce *Mdde* from 3 to 48 h, and the peak appeared at 36–48 h post infection, whereas injection of PGN-EB could only strongly induced *BdPho* at 3 and 6 h post challenge, and the peak appeared at 6 h post injection. The differences might be due to injection of massive pure PGN-EB could induce *BdPho* quickly while injection of *E. coli* required the expansion of bacteria to induce *BdPho*. It might also be due to different responses of innate immune system to *E. coli* (containing PGN and some other components) and PGN-EB.

Mating is considered as an indispensable physiological process in insects, and immunity is the basic aspect for individual survival. Thus, both immunity and mating are closely linked to fitness, and several studies reported the interactions between these two processes (Lawniczak et al. 2007). As previously reported, mating can have two different effect on immunity: either suppress or increase immunity. It was reported that there were tradeoffs between reproduction and immunity, and an increase in reproduction is generally coupled with a decrease in immunity (Fedorka et al. 2007). Mating leading to a reduction of immunity has been reported in *Tenebrio molitor* (Rolff and Siva-Jothy 2002), *Atta colombica* (Baer et al. 2006) and *Matrona basilaris japonica* (Siva-Jothy et al. 1998). However, some evidence suggested that immunity of insect might also increase after mating. For example, mating resulting in enhancement of immunity has been reported in *Gryllus texensis* (Shoemaker et al. 2006) and *D. melanogaster* (Domanitskaya et al. 2007; Peng et al. 2005). In this study, the results found that mating enhanced the immunity in *B. dorsalis* by up-regulation of the AMP gene such as *BdPho*, which might protect the host from immune challenge during mating. This up-regulation of AMP gene may result from transfer of sperm and male accessory protein such as sex-peptide to the female during mating (Lawniczak and Begun 2004; Peng et al. 2005).

Taken all together, we cloned and characterized a phormicin gene from *B. dorsalis*, and investigated the spatiotemporal expression patterns of *BdPho*. Furthermore, we determined the mRNA levels of *BdPho* in female adults in response to peptidoglycan challenge and mating stimulation, which lays the foundation for understanding the molecular mechanisms in immune system and may help us to make use of insect pathogens for pest control. Furthermore, the basic knowledge derived from this study may provide information for extension the source of novel antibiotics.

## Additional files

**Additional file 1: Figure S1.** Stability validation of *RPS3* in this study. A, stability of *RPS3* in different developmental stages and adult tagmata in *B. dorsalis*. E: eggs; L: larvae; P: pupae; A: adults; He: head; Th: thorax; Ab: abdomen. B, stability of *RPS3* in different tissue of adult, mated female and virgin female. Mg: mid gut; FB: fat body; MT: Malpighian tubule; Ov: ovary; Te: testis; MF: mated female; VM: virgin female. C, stability of RPS3 after PGN challenge. EB: PGN-EB; SA: PGN-SA; W: sterile endotoxin-free water; CK: not injected.

**Additional file 2: Figure S2.** Distribution of Cq values of *α*-*tublin* and *RPS3* obtained using qRT-PCR.

## Abbreviations

AMP: antimicrobial peptide
Pho: phormicin
PGN: peptidoglycan
PGN-EB: peptidoglycans from *Escherichia coli* O111:B4
PGN-SA: peptidoglycans from *Staphylococcus aureus*
RH: relative humidity
RNA: ribonucleic acid
cDNA: complementary deoxyribonucleic acid
PCR: polymerase chain reaction
DNase: deoxyribonuclease
qRT-PCR: quantitative real-time PCR
ANOVA: analysis of variance
RT-PCR: reverse transcription PCR
