# Supplementary figures and images for: Antimicrobial peptide gene BdPho responds to peptidoglycan infection and mating stimulation in oriental fruit fly, Bactrocera dorsalis (Hendel)
Source: AMB Express. 2018 Jan 11;8:5. doi: 10.1186/s13568-017-0533-8 (PMC5764898; doi:10.1186/s13568-017-0533-8)

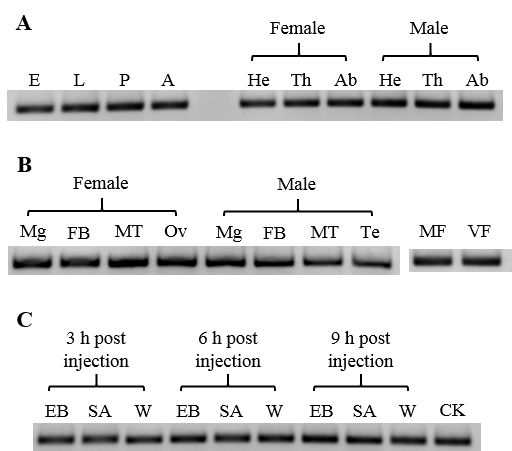

Supplement: Supplementary file 1 — Additional file 1: Figure S1. Stability validation of RPS3 in this study. A, stability of RPS3 in different developmental stages and adult tagmata in B. dorsalis. E: eggs; L: larvae; P: pupae; A: adults; He: head; Th: thorax; Ab: abdomen. B, stability of RPS3 in different tissue of adult, mated female and virgin female. Mg: mid gut; FB: fat body; MT: Malpighian tubule; Ov: ovary; Te: testis; MF: mated female; VM: virgin female. C, stability of RPS3 after PGN challenge. EB: PGN-EB; SA: PGN-SA; W: sterile endotoxin-free water; CK: not injected. [file 13568_2017_533_MOESM1_ESM.tif]

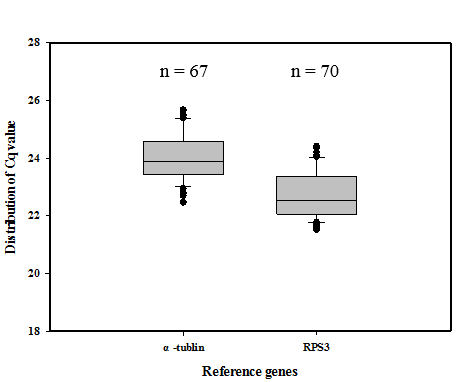

Supplement: Supplementary file 2 — Additional file 2: Figure S2. Distribution of Cq values of α-tublin and RPS3 obtained using qRT-PCR. [file 13568_2017_533_MOESM2_ESM.tif]
